# Predictors of survival and renal replacement therapy in anca-associated vasculitis with renal involvement: An 11-year real-world cohort from North Macedonia

**DOI:** 10.1016/j.clinsp.2026.101126

**Published:** 2026-09-09

**Authors:** Vlatko Karanfilovski, Nikola Gjorgjievski, Angela Kabova, Zhaklina Shterjova-Markovska, Mimoza Milenkova, Pavlina Dzekova-Vidimliski, Igor Nikolov, Gjulsen Selim, Slavica Kostadinova Kunovska, Biljana Gerasimovska Kitanovska

**Affiliations:** a University Clinic for Nephrology, Faculty of Medicine, University Ss. Cyril and Methodius, Skopje, Republic of North Macedonia; b Institute of Pathology Medical Faculty Skopje, Skopje, Republic of North Macedonia

**Keywords:** ANCA-associated vasculitis, Kidney diseases, North Macedonia, Prognosis, Survival analysis

## Abstract

**Background:**

Antineutrophil Cytoplasmic Antibody (ANCA) – Associated Vasculitis (AAV) is a rare systemic small-vessel vasculitis in which renal involvement is a major determinant of morbidity and survival. Despite therapeutic advances, many patients still present with advanced kidney disease. Data from Southeast Europe remain limited.

**Methods:**

We conducted an 11-year retrospective observational study of adult patients with AAV and renal involvement treated at national nephrology center in North Macedonia. Demographic, clinical, laboratory, treatment, and outcome data were analyzed. Predictors of mortality were evaluated using Cox proportional hazards models, while renal outcomes were assessed using Fine-Gray competing risk regression with death treated as a competing event.

**Results:**

Forty-two patients were included (57.1% male; mean age 56.5 ± 14.9 years). Renal involvement was severe at presentation, with a mean eGFR of 26.2 ± 16.4 mL/min/1.73 m^2^ corresponding to CKD stage G4. Pulmonary involvement was observed in 71.4%, whereas 21.4% had renal-limited disease. During follow-up, 27 patients (64.3%) progressed to RRT and 17 (40.5%) died. Median overall survival was 84.0-months (95% CI 57.4–110.6). In multivariable Cox regression, older age independently predicted mortality (HR = 1.05, 95% CI 1.01–1.10). In multivariable Fine-Gray competing risk analysis, lower baseline eGFR independently predicted progression to ESRD (sHR 0.96 per/mL/min/1.73 m^2^ increase, 95% CI 0.93–0.99), whereas pulmonary involvement was not independently associated with either outcome.

**Conclusion:**

This first national cohort from North Macedonia confirms that AAV with renal involvement presents with advanced kidney dysfunction, a compressed time to RRT, and substantial early mortality. Baseline renal function remains the dominant determinant of renal outcome, underscoring the need for earlier diagnosis and structured referral pathways.

## Highlights

• AAV presents with advanced renal dysfunction at diagnosis.
• High rates of progression to renal replacement therapy observed.
• Early mortality remains substantial in real-world cohort.
• Lower baseline eGFR strongly predicts progression to ESRD.
• Older age independently associated with increased mortality.

## Introduction

Anti-Neutrophil Cytoplasmic Antibody (ANCA) – Associated Vasculitis (AAV) represents a group of rare, potentially life-threatening systemic small-vessel necrotizing vasculitis, including Granulomatosis with Polyangiitis (GPA), Mcroscopic Polyangiitis (MPA), and Eosinophilic Granulomatosis with Polyangiitis (EGPA). . ^1^ Renal involvement in the form of rapidly progressive pauci-immune glomerulonephritis is one of the most severe and prognostically relevant manifestations of AAV and remains a major cause of Chronic Kidney Disease (CKD), End-Stage Renal Disease (ESRD), and excess mortality. ^2^

Before the introduction of immunosuppressive therapy, the prognosis of AAV was dismal; however, the advent of glucocorticoids and cytotoxic agents, followed by the incorporation of targeted therapies such as rituximab, has substantially improved short- and medium-term outcomes. ^3^ Despite these advances, contemporary cohort studies consistently demonstrate that a considerable proportion of patients still present with advanced renal impairment at diagnosis, and up to one-third-ultimately require Renal Replacement Therapy (RRT). ^4^^,^^5^ Moreover, patients with AAV continue to experience significantly higher mortality compared with the general population, particularly during the early phase following diagnosis. ^4^

Disease heterogeneity further complicates risk stratification in AAV. Traditional classification based on clinical phenotype (GPA, MPA, EGPA) often overlaps, whereas classification according to ANCA specificity ‒ Proteinase-3 (PR3)-ANCA versus Myeloperoxidase (MPO)-ANCA ‒ has emerged as a biologically and prognostically meaningful framework. ^6^ Serological classification has been shown to better predict relapse risk and clinical course, with PR3-ANCA-positive disease generally associated with a more aggressive and relapsing phenotype, while MPO-ANCA-positive disease is more frequently linked to severe renal involvement and progression to kidney failure. ^7^^,^^8^

Large prospective trials and registry-based studies, particularly those conducted by the European Vasculitis Society (EUVAS), have identified older age, impaired renal function at diagnosis, and pulmonary involvement as key predictors of adverse outcomes in ANCA-associated vasculitis. ^4^^,^^5^ However, long-term real-world data remain limited, and marked geographic variability persists, with scarce data from Southeast Europe and the Western Balkans. ^8^

In this context, we hypothesized that, in a national real-world cohort of patients with ANCA-associated vasculitis and renal involvement, baseline renal function is the principal determinant of renal outcomes and survival. To test this hypothesis, we conducted the first comprehensive 11-year cohort study in North Macedonia.

## Materials and methods

Ethical approval was obtained from the Institutional Review Board of the University Hospital of Nephrology, Skopje, Republic of North Macedonia (protocol number: 01–02/2026). This approval was granted retrospectively for analysis of anonymized clinical data collected during routine clinical care, in accordance with national regulations governing observational studies. Given the retrospective nature of the study, the requirement for individual informed consent was waived. All patient data were anonymized and handled in compliance with data protection regulations.

An observational, retrospective, single-center study was carried out over a period of 11-years (from year 2014 to year 2025) on a group of patients older than 14-years who were hospitalized and treated at University Hospital of Nephrology in Skopje, Republic of North Macedonia. This institution is a tertiary care hospital and the leading national referral center for nephrology, particularly for glomerular diseases and systemic disorders with renal involvement with >2500 hospitalizations per year.

The study included: 1) Patients with histopathological confirmed pauci-immune small-vessel vasculitis on kidney biopsy and positive serum Myeloperoxidase (MPO) – or Proteinase-3 (PR3) – ANCA antibodies; 2) Patients with a clinical presentation consistent with ANCA-associated vasculitis and positive MPO- or PR3-ANCA serology without kidney biopsy; and 3) Patients with histopathological confirmed pauci-immune small-vessel vasculitis on kidney biopsy but negative ANCA serology, classified as ANCA-negative vasculitis.

Patients with biopsy-proven pauci-immune glomerulonephritis and negative ANCA serology were included because ANCA-negative vasculitis represents a recognized clinical phenotype within the spectrum of ANCA-associated vasculitis that is encountered in routine clinical practice. A sensitivity analysis confirmed that their inclusion did not materially affect the findings. Serum MPO-ANCA and PR3-ANCA antibodies were measured using standardized enzyme-linked immunosorbent assay (ELISA; EUROIMMUN, Lübeck, Germany), performed according to the manufacturer’s instructions at the Institute of Immunobiology and Human Genetics in Skopje, with internal quality controls routinely applied to ensure analytical validity. Renal biopsy was performed under ultrasound guidance at the University Hospital of Nephrology in Skopje, and the histopathological evaluation of biopsy specimens was conducted at the Institute of Pathology in Skopje by two independent pathologists with expertise in field of nephropathology.

Our research was based on the analysis of data from medical histories, national e-Health System “Moj Termin” and hospital discharge tickets of the patients. We compiled data on epidemiological characteristics, comorbidities, clinical presentation, and laboratory parameters, including 24-hour proteinuria, presence of hematuria, and serum creatinine at the time of diagnosis and at the initiation of RRT, as well as on treatment strategy and patient outcomes. The estimated glomerular filtration rate was calculated using the Chronic Kidney Disease Epidemiology Collaboration Equation (CKD-Epi), which takes into account serum creatinine concentration, age, and sex. ^9^ Rheumatologic manifestations were defined as musculoskeletal symptoms attributable to ANCA-associated vasculitis, including arthralgia, arthritis, and myalgia, in the absence of alternative causes. Pulmonary manifestations were defined as upper and/or lower respiratory tract involvement, confirmed clinically by symptoms such as cough, dyspnea, hemoptysis, or sinusitis, and/or radiologically by chest radiography or computed tomography findings including pulmonary infiltrates, nodules, ground-glass opacities, or features of diffuse alveolar hemorrhage. Cutaneous manifestations were defined as skin involvement attributed to small-vessel vasculitis, including palpable purpura, petechiae, ulcers, or livedo reticularis, in the absence of alternative causes. Renal-limited ANCA-associated vasculitis was defined as disease confined to the kidneys, characterized by rapidly progressive glomerulonephritis with no clinical, radiological, or histopathological evidence of involvement of other organ systems. ^10^

Renal replacement therapy was initiated based on standard clinical criteria, including hyperkalemia, metabolic acidosis, volume overload with pulmonary edema, or uremic symptoms, rather than a predefined eGFR threshold. Hemodynamically stable patients underwent standard intermittent hemodialysis with unfractionated heparin or low–molecular-weight heparin anticoagulation. Patients who were hemodynamically unstable and developed diffuse alveolar hemorrhage were treated with continuous veno-venous hemodiafiltration with continuous heparinization. Induction therapy consisted of pulse methylprednisolone 1 g daily for three consecutive days followed by tapering, combined with intravenous cyclophosphamide (15 mg/kg, adjusted for age and estimated glomerular filtration rate) or rituximab 1 g on days 0 and 14, administered in accordance with contemporaneous Kidney Disease: Improving Global Outcomes (KDIGO) Clinical Practice Guidelines for the management of antineutrophil cytoplasmic antibody–associated vasculitis .^11^, ^12^, ^13^ Therapeutic plasma exchanges was performed in patients with life-threatening manifestations, including severe diffuse alveolar hemorrhage with respiratory failure or rapidly progressive renal decline requiring RRT. In this study, End-Stage Renal Disease (ESRD) was operationally defined as the initiation of Renal Replacement Therapy (RRT), as all patients who progressed to ESRD ultimately required RRT. Accordingly, the terms ESRD and RRT are used synonymously when referring to this renal outcome. Renal survival was defined as the time from diagnosis to initiation of RRT, and patient survival as the time from diagnosis to death.

Continuous variables were tested for normality using the Shapiro-Wilk test and are presented as mean ± standard deviation or median with interquartile range, as appropriate. Categorical variables are presented as counts and percentages and were compared using the χ^2^ test or Fisher’s exact test, as appropriate. Overall patient survival was analyzed using Kaplan-Meier methods, with death as the event of interest, and survival curves were compared using the log-rank test. Independent predictors of mortality were evaluated using Cox proportional hazards regression models. Hazard Ratios (HRs) with 95% Confidence Intervals (95% CIs) were calculated. The proportional hazards assumption was assessed using Schoenfeld residuals. The results of the Schoenfeld residual tests are presented in Supplementary Table 1. Given the limited number of mortality events (n = 17), the multivariable Cox model was restricted to two prespecified clinically relevant covariates to reduce the risk of overfitting. Progression to ESRD was analyzed within a competing risk framework, with death consistently treated as a competing event in all renal outcome analyses. Cumulative incidence functions were estimated and compared between groups using Gray’s test. Multivariable analysis for renal outcomes was performed using Fine-Gray subdistribution hazard models, and subdistribution Hazard Ratios (sHRs) with 95% Confidence Intervals were reported. Statistical analyses were performed using SPSS Statistics version 17 (IBM Corp., Armonk, NY, USA) and R software version 4.5.2 (R Foundation for Statistical Computing, Vienna, Austria), with the survival and cmprsk packages. A p-value < 0.05 was considered statistically significant.

This study was conducted and reported in accordance with the Strengthening the Reporting of Observational Studies in Epidemiology (STROBE) statement.

## Results

### Baseline characteristics of the study population

A total of 42 patients with a definitive diagnosis of ANCA-associated vasculitis and renal involvement were enrolled in the study. Of these, 24 patients (57.1%) were male, with a mean age at diagnosis of 56.5 ± 14.9 years. Common comorbidities included hypertension in 31 patients (73.8%), diabetes mellitus in 9 patients (21.4%), cardiac disease at diagnosis in 7 patients (16.7%), and no cases of malignancy.

Regarding ANCA type, 5 patients (11.9%) were ANCA-negative, 18 patients (42.9%) were cANCA (PR3-ANCA) ‒ positive, and 19 patients (45.2%) were pANCA (MPO-ANCA) ‒ positive. Other organ involvement included rheumatologic manifestations in 20 patients (47.6%), cutaneous manifestations in 6 patients (14.3%), and pulmonary manifestations in 30 patients (71.4%). Nine patients (21.4%) had renal-limited ANCA-associated vasculitis.

The majority of the patients in our cohort presented with advanced renal impairment at the time of diagnosis, with a mean serum creatinine level of 282.3 ± 169.6 µmoL/L and a mean eGFR of 26.2 ± 16.4 mL/min; notably, 29 patients (69.0%) had an eGFR below 30 mL/min, indicating severe renal involvement. Renal biopsy was performed in 30 patients (71.4%) (Table 1).

**Table 1:** Baseline characteristics, clinical features, and treatment of the study population (n = 42).

Variable	Value / n (%)	Mean ± SD / Median [IQR]
Demographics		
Age in time of diagnosis (years)	–	56.5 ± 14.9
Sex (male)	24 (57.1%)	–
**ANCA type**		
ANCA negative	5 (11.9%)	–
cANCA	18 (42.9%)	–
pANCA	19 (45.2%)	–
**Renal features**		
Proteinuria g/L		1.68 ± 1.0 g/L
Proteinuria > 1 *g*/day	28 (71.8%)	
Hematuria	42 (100%)	–
eGFR at diagnosis (CKD-EPI, mL/min/1.73 m^2^)	–	26.2 ± 16.4
Creatinine at diagnosis (µmoL/L)	–	282.3 ± 169.6
AAV renal-limited	9 (21.4%)	–
RRT required during follow-up	27 (64.3%)	–
eGFR at RRT initiation (mL/min/1.73 m^2^)	–	10.1 ± 3.6
Time on RRT (months)^a^	–	33.5 ± 27.5
**Treatment**		
Induction: Steroid pulses	42 (100%)	–
Induction: Cyclophosphamide	29 (69.0%)	–
Induction: Rituximab	4 (9.5%)	–
Maintenance: Oral steroids	32 (78.0%)	–
Maintenance therapy: Oral cyclophosphamide	11 (26.8%)	–
Maintenance therapy: Rituximab	4 (9.8%)	–
Maintenance therapy: MMF / Tacrolimus / Azathioprine	7 (17.1%)	–
**Outcomes**		
Death (any cause)	17 (40.5%)	–
Death not due to AAV related cause	4 (25.0%)	–
Death within 3-years from diagnosis	11 (26.2%)	–
Follow-up time (months)	–	30.0 ± 31.1

a Time on RRT represents the duration of renal replacement therapy among patients who initiated RRT (n = 27), calculated from RRT initiation to the end of follow-up or death.

### Factors associated with overall survival

Baseline characteristics stratified according to survival status are presented in Table 2. Deceased patients were significantly older at diagnosis compared with survivors (60.1 ± 10.7 vs. 50.8 ± 16.7 years; 95% CI for the difference, 0.7–17.8 years; p = 0.034). Pulmonary involvement was more frequent among non-survivors than survivors (88.2%vs. 60.0%, p = 0.047), whereas renal-limited disease was more common among survivors (32.0%vs. 5.9%, p = 0.043). Mortality proportions differed across ANCA subtypes (p = 0.046). Baseline renal function and proteinuria did not significantly differ between groups. In univariate Cox proportional hazards analysis, older age was associated with increased mortality (HR = 1.05 per year, 95% CI 1.01–1.10, p = 0.021). In the multivariable Cox model, restricted to age and pulmonary involvement to avoid overfitting, age remained independently associated with mortality (HR = 1.05, 95% CI 1.01–1.10, p = 0.021), while pulmonary involvement showed a non-significant trend toward higher mortality risk (HR = 3.57, 95% CI 0.81–15.73, p = 0.093) (Table 3).

**Table 2:** Baseline characteristics in patients with ANCA-associated vasculitis according to survival status.

Variables	Survivors (n = 25)	Deceased (n = 17)	p
	n (%)	n (%)	
**ANCA type**			0.046
ANCA negative	3 (12%)	2 (11.8%)	
PR3 – ANCA	7 (28%)	11 (64.7%)	
MPO – ANCA	15 (60%)	4 (23.5%)	
**Pulmonary manifestations**	15 (60%)	15 (88.5%)	0.047
**AAV renal-limited**	8 (32.0%)	1 (5.9%)	0.043
**Age (years)**	50.8 ± 16.7	60.1 ± 10.7	0.034
**Proteinuria (g/L)**	1.83 ± 1.69	2.36 ± 1.71	0.391
**eGFR at diagnosis (mL/min/1.73m^2^)**	22.1 ± 18.9	24.2 ± 14.6	0.683

**Table 3:** Multivariable Cox proportional hazards model for overall mortality.

Variable	HR	95% CI	p-value
Age (per year)	1.05	1.01–1.10	0.021
Pulmonary involvement	3.57	0.81–15.73	0.093

HR, Hazard Ratio; CI, Confidence Interval.

Multivariable model adjusted for age and pulmonary involvement.

### Factors associated with renal survival

Baseline characteristics stratified according to progression to RRT are presented in Table 4. Patients who progressed to RRT during follow-up had more severe renal impairment at presentation, with significantly higher baseline serum creatinine levels (472.4 ± 307.2 vs. 249.1 ± 119.6 µmoL/L; 95% CI for the difference, 88.1–358.4 µmoL/L; p = 0.002) and lower baseline eGFR (17.8 ± 15.1 vs. 32.3 ± 16.9 mL/min/1.73 m^2^; 95% CI for the difference, 3.8–25.3 mL/min/1.73 m^2^; p = 0.010) compared with patients who remained dialysis-free. The prevalence of diabetes mellitus was higher among patients who progressed to RRT, although this difference did not reach statistical significance (29.6%vs. 6.7%, p = 0.082). No statistically significant differences were observed for the remaining baseline characteristics.

**Table 4:** Baseline characteristics in patients with ANCA-associated vasculitis according to progression to RRT.

Variable	No RRT (n = 15)	RRT Required (n = 27)	p
Diabetes mellitus	1 (6.7%)	8 (29.6%)	0.082
Renal-limited AAV	5 (33.3%)	4 (14.8)	0.167
Age (years)	49.3 ± 18.1	57.5 ± 12.7	0.135
eGFR at diagnosis (mL/min/1.73m^2^)	32.3 ± 16.9	17.8 ± 15.1	0.010

### Overall survival

Kaplan-Meier analysis of the entire cohort demonstrated a median overall survival of 84.0 months (95% CI: 57.4–110.6). Overall survival declined rapidly during early follow-up, with a substantial proportion of deaths occurring within the first year after diagnosis, followed by a more stable survival trajectory thereafter.

**Fig. 1:**
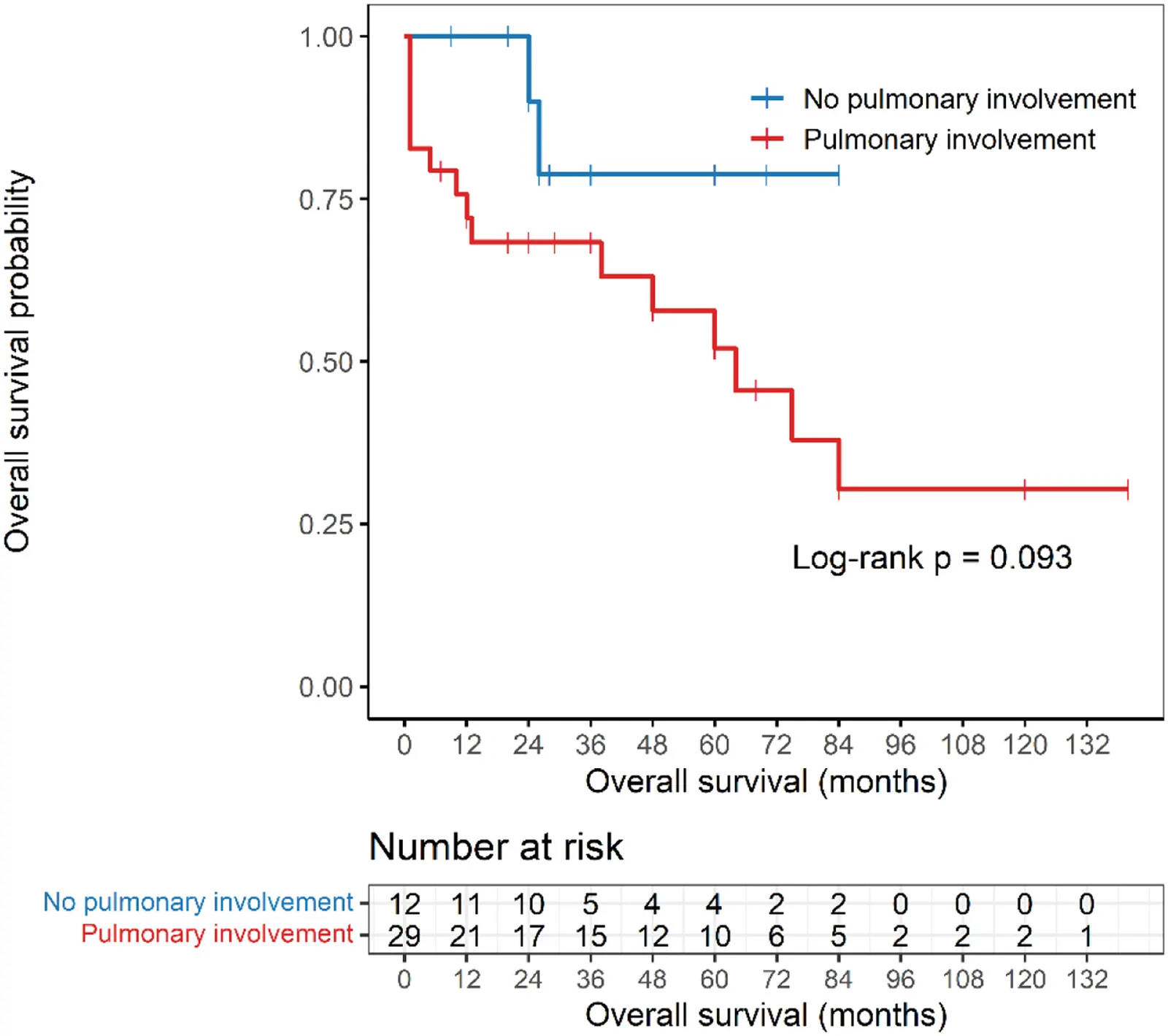
Kaplan-Meier overall survival according to pulmonary involvement. Patients with pulmonary involvement had numerically lower survival; however, the difference was not statistically significant (log-rank p = 0.093).

Kaplan-Meier analysis stratified by pulmonary involvement showed lower survival among patients with pulmonary manifestations; however, this difference did not reach statistical significance (log-rank p = 0.093) (Fig. 1).

### Renal outcomes and progression to renal replacement therapy

**Fig. 2:**
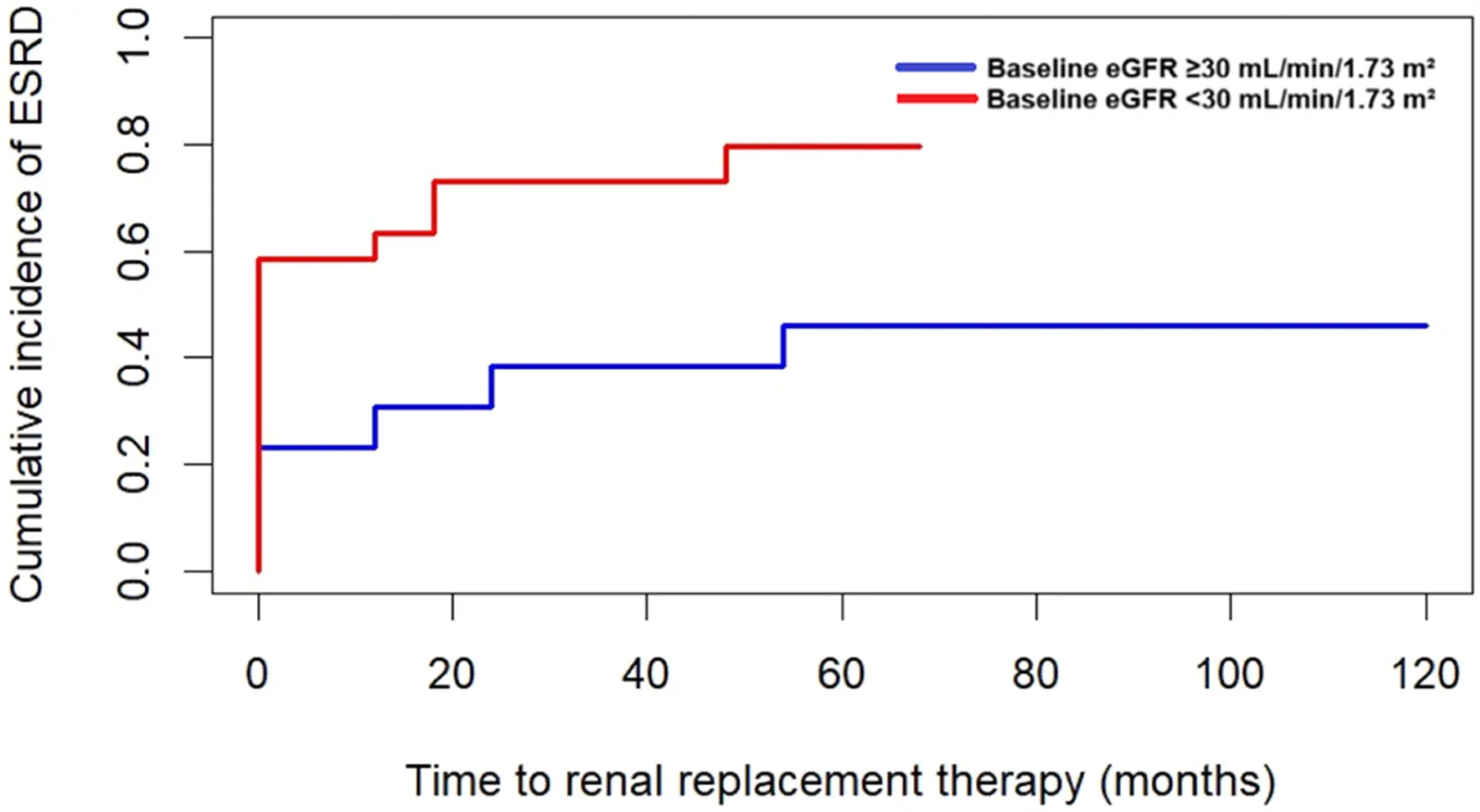
Cumulative incidence of progression to end-stage renal disease (ESRD) according to baseline eGFR category. Cumulative incidence functions were estimated using a competing risk approach, with death treated as a competing event (Gray’s test, p = 0.028).

In competing risk analysis, lower baseline eGFR was associated with a significantly higher cumulative incidence of progression to ESRD (Gray’s test, p = 0.028, Fig. 2).

In the multivariable Fine–Gray competing risk model, lower baseline eGFR remained independently associated with progression to ESRD (sHR = 0.96 per/mL/min/1.73 m^2^ increase, 95% CI 0.93–0.99, p = 0.009). Age and pulmonary involvement were not independently associated with ESRD risk (Table 5).

**Table 5:** Multivariable fine-gray competing risk model for progression to ESRD.

Variable	sHR	95% CI	p-value
eGFR (per mL/min/1.73 m^2^)	0.96	0.93–0.99	0.009
Age	1.01	0.99–1.03	0.39
Pulmonary involvement	1.35	0.78–2.31	0.28

sHR, Subdistribution Hazard Ratio; CI, Confidence Interval.

Model adjusted for age and pulmonary involvement.

Model includes n = 42 patients; ESRD events = 27; competing deaths before ESRD = 5.

A sensitivity analysis excluding the five ANCA-negative patients yielded materially similar results. Baseline eGFR remained independently associated with progression to ESRD (sHR = 0.96, 95% CI 0.92–0.99, p = 0.019), while age and pulmonary involvement were not significant predictors.

## Discussion

This study provides the first comprehensive 11-year real-world analysis of ANCA-associated vasculitis with renal involvement in North Macedonia, offering region-specific data from an understudied Southeast European population and confirming previously established prognostic associations in this setting. The relatively modest sample size, inherent to the rarity of ANCA-associated vasculitis, limits the precision of some effect estimates and precludes more extensive multivariable adjustment. Nevertheless, the consistency of our findings with larger international cohorts supports the validity of the observed associations. Patients in our cohort presented with markedly advanced renal disease at diagnosis, reflected by a mean eGFR of 26.2 ± 16.4 mL/min/1.73 m^2^ and 69% having eGFR < 30 mL/min/1.73 m^2^. This severity surpasses that reported in many Western cohorts, where baseline renal impairment is generally less advanced upon presentation.^4^^,^^14^ In our cohort, pulmonary involvement was observed in 71% of patients, whereas renal-limited disease accounted for only 21%, highlighting a predominance of systemic disease at diagnosis.

Survival outcomes in our cohort were substantially poorer compared with contemporary literature. Although Kaplan-Meier analysis demonstrated a relatively long median overall survival of 84-months, survival declined rapidly during the early follow-up period, with a substantial proportion of deaths occurring within the first year after diagnosis. This contrasts with EUVAS and large registry-based cohorts reporting one-year survival between 82% and 93% and five-year survival rates of 76%–88%.^4^^,^^14^, ^15^, ^16^ The discrepancy between relatively long median survival and poor early outcomes reflects excess mortality concentrated in the first year after diagnosis, followed by a more stable survival trajectory. This pattern likely relates to delayed diagnosis and advanced renal dysfunction at presentation rather than intrinsic biological differences. Supporting this interpretation, smaller cohorts such as that reported by Dadonienė et al. (2017) demonstrated more favorable survival outcomes in less severely affected populations.^17^

In univariate Cox regression analysis, older age was associated with higher mortality. Pulmonary involvement and PR3-ANCA positivity were more frequent among non-survivors in baseline comparisons, whereas renal-limited AAV was more common among survivors. In multivariable Cox models restricted to clinically relevant covariates, only older age remained independently associated with mortality, with an approximately 5% higher risk of death per additional year of age (HR = 1.05, 95% CI 1.01–1.10). These observations are broadly consistent with prior cohorts demonstrating that age is a major determinant of survival in ANCA-associated vasculitis, whereas organ involvement often reflects overall disease burden rather than serving as an independent driver of mortality.^18^ In this context, our results confirm patterns reported in international studies rather than identifying novel mortality predictors.

Renal outcomes in our cohort were predominantly determined by baseline renal function. In multivariable Fine-Gray competing risk models, higher baseline eGFR was independently associated with a lower risk of progression to RRT, corresponding to an approximately 4% reduction in ESRD risk per 1 mL/min/1.73 m^2^ increase (sHR = 0.96, 95% CI 0.93–0.99), whereas ANCA type and pulmonary involvement were not independently associated with ESRD. These findings are consistent with prior studies demonstrating that baseline renal function and histological severity outweigh ANCA specificity in predicting renal survival.^18^ Although the overall incidence of ESRD was comparable to long-term EUVAS data, [14,19] progression to RRT occurred earlier in our cohort, suggesting a compressed renal trajectory in the setting of advanced disease at diagnosis. Using a competing risk framework that accounted for death as a competing event, patients with baseline eGFR < 30 mL/min/1.73 m^2^ had a significantly higher cumulative incidence of ESRD, underscoring the importance of appropriate statistical methodology in cohorts with substantial early mortality.^20^

A considerable proportion of our patients required dialysis at or shortly after diagnosis, a subgroup known to have limited renal recovery. Previous reports indicate that many such patients remain dialysis-dependent despite appropriate induction therapy.^21^^,^^22^ Nevertheless, immunosuppressive treatment remains essential to control systemic disease and prevent life-threatening extra-renal manifestations.^22^

Pulmonary involvement, frequently associated with PR3-ANCA and granulomatosis with polyangiitis, has been linked to worse outcomes in multiple cohorts.^23^ In our study, pulmonary manifestations were more common among non-survivors in baseline comparisons and were associated with higher mortality in univariate Cox analysis; however, they did not retain independent prognostic significance in multivariable models. This suggests that pulmonary involvement may reflect overall disease burden rather than act as an independent determinant of mortality. Similar patterns have been reported in cohorts from neighboring regions, including Türkiye, where pulmonary and renal involvement were major contributors to adverse outcomes.^24^ The paucity of large, well-characterized cohorts from the Balkan region underscores a significant gap in the literature and highlights the importance of national real-world analyses.^25^

This study’s strengths lie in its comprehensive national coverage over an extended period and detailed capture of clinical, serological, and outcome data, providing a real-world perspective rarely available from Southeast Europe. Limitations include the relatively small sample size inherent to rare diseases and potential referral bias toward patients with severe renal involvement. One patient was younger than 18-years at diagnosis (16-years), which may introduce minimal age-related heterogeneity, although its impact on the overall results is negligible. Incomplete histopathological data limited the systematic application of validated biopsy-based prognostic classifications, such as the Renal Risk Score and Berden classification. Indirect Immunofluorescence (IIF) testing was not systematically recorded and have been performed only in selected cases, which could have led to potential misclassification of a subset of ANCA-negative patients. As histopathological features provide important prognostic information, future prospective studies incorporating standardized biopsy assessment may further refine risk stratification in this population. The relatively small cohort size and limited number of mortality events (n = 17) restrict the statistical power of multivariable mortality analyses. Although the multivariable Cox model was intentionally restricted to clinically relevant covariates to reduce the risk of overfitting, the findings related to mortality should be interpreted cautiously. Because proteinuria was not included in the multivariable model due to event count constraints, we cannot exclude the possibility that proteinuria provides additional prognostic information beyond eGFR. Treatment allocation was not randomized and likely influenced by disease severity and clinical presentation. Rituximab became available for nephrological indications only in recent years and was therefore not used throughout the entire study period. The majority of patients presented with advanced renal dysfunction, in which cyclophosphamide remains the preferred induction therapy according to KDIGO recommendations (11,12,13), while rituximab was used selectively in patients with less severe disease. This treatment heterogeneity, with cyclophosphamide preferentially administered to patients with more advanced renal dysfunction and rituximab generally reserved for patients with less severe disease, may have introduced indication bias and residual confounding. Consequently, treatment-outcome associations should not be interpreted as comparative estimates of therapeutic efficacy. Despite these limitations, the consistency of our findings across multiple analytical approaches strengthens the validity of the observed associations.

In conclusion, this first national cohort from North Macedonia confirms that ANCA-associated vasculitis with renal involvement presents with advanced kidney dysfunction and substantial early mortality. The compressed time to renal replacement therapy observed in our cohort reflects delayed diagnosis and referral rather than fundamentally different disease biology. Baseline renal function emerged as the dominant determinant of progression to ESRD, consistent with international evidence. These findings underscore the need for region-specific strategies aimed at earlier recognition, streamlined diagnostic pathways, and timely referral in order to reduce preventable renal failure and early mortality in this population.

## Authors’ contributions

Vlatko Karanfilovski: Collected the data; Participated in statistical analysis, and drafted the manuscript. Nikola Gjorgjievski, Angela Kabova, Zhaklina Shterjova-Markovska, and Mimoza Milenkova: Contributed to data collection and interpretation. Pavlina Dzekova-Vidimliski, Igor Nikolov, Gjulsen Selim, Slavica Kostadinova Kunovska, and Biljana Gerasimovska Kitanovska: Contributed to statistical interpretation; Provided critical revisions, and offered scientific suggestions. All authors reviewed and approved the final manuscript.

## Funding

This research did not receive any specific grant from funding agencies in the public, commercial, or not-for-profit sectors.

## Conflict of interest

The authors declare no conflicts of interest.

## Data Availability

The data that support the findings of this study are available from the corresponding author upon reasonable request. Due to ethical restrictions and patient confidentiality, the data are not publicly available.
