# Supplementary material for: Predictors of survival and renal replacement therapy in anca-associated vasculitis with renal involvement: An 11-year real-world cohort from North Macedonia
Source: Clinics (Sao Paulo). 2026 Sep 9;81:101126. doi: 10.1016/j.clinsp.2026.101126 (PMC13573694; doi:10.1016/j.clinsp.2026.101126)
Supplement: Supplementary file 1 [file mmc2.pdf]

Ethics in publishing

1. Does your research involve experimentation on animals?:
No

2. Does your study include human subjects?:
Yes

- If yes; please provide name of the ethical committee approving these experiments and the registration number. :
  The study was conducted in accordance with the Declaration of Helsinki. Ethical approval was obtained from the Institutional Review Board of the University Hospital of Nephrology, Skopje, Republic of North Macedonia (No 07/5-26). Given the retrospective nature of the study, the requirement for individual informed consent was waived. All patient data were anonymized and handled in compliance with data protection regulations.
- If yes; please confirm authors compliance with all relevant ethical regulations. :
  Yes
- If yes; please confirm that written consent has been obtained from all patients. :
  Yes

3. Does your study include a clinical trial?:
No

4. Are all data shown in the figures and tables also shown in the text of the Results section and discussed in the Conclusions?:
Yes
