# Supplementary material for: Predictors of survival and renal replacement therapy in anca-associated vasculitis with renal involvement: An 11-year real-world cohort from North Macedonia
Source: Clinics (Sao Paulo). 2026 Sep 9;81:101126. doi: 10.1016/j.clinsp.2026.101126 (PMC13573694; doi:10.1016/j.clinsp.2026.101126)
Supplement: Supplementary file 2 [file mmc1.docx]

**CLINICS-D-26-00303_ Supplementary Material**

**Supplementary Table 1** Assessment of proportional hazards assumption using Schoenfeld residuals.

| **Variable** | **p-value** |
| --- | --- |
| Age | 0.66 |
| Pulmonary involvement | 0.59 |
| Global test | 0.81 |
